# Neurofilament levels, disease activity and brain volume during follow-up in multiple sclerosis

**DOI:** 10.1186/s12974-018-1249-7

**Published:** 2018-07-18

**Authors:** Irene Håkansson, Anders Tisell, Petra Cassel, Kaj Blennow, Henrik Zetterberg, Peter Lundberg, Charlotte Dahle, Magnus Vrethem, Jan Ernerudh

**Affiliations:** 1 Department of Neurology and Department of Clinical and Experimental Medicine, Linköping University, Linköping, Sweden; 2 Radiation Physics, Department of Medical and Health Sciences, Linköping University, Linköping, Sweden; 3 Center for Medical Image Science and Visualization (CMIV), Linköping University, Linköping, Sweden; 4 Department of Clinical Immunology and Transfusion Medicine and Department of Clinical and Experimental Medicine, Linköping University, Linköping, Sweden; 5 Inst. of Neuroscience and Physiology, Department of Psychiatry and Neurochemistry, The Sahlgrenska Academy at the University of Gothenburg, Gothenburg, Sweden; 6 Clinical Neurochemistry Laboratory, Sahlgrenska University Hospital, Mölndal, Sweden; 7 Department of Molecular Neuroscience, UCL Institute of Neurology, Queen Square, London, UK; 8 UK Dementia Research Institute at UCL, London, UK; 9 Radiology, Department of Medical and Health Sciences, Linköping University, Linköping, Sweden

**Keywords:** Multiple sclerosis, Clinically isolated syndrome, Disease activity, Neurofilament light chain, CHI3L1, CXCL10, Brain volume

## Abstract

**Background:**

There is a need for clinically useful biomarkers of disease activity in clinically isolated syndrome (CIS) and relapsing remitting MS (RRMS). The aim of this study was to assess the correlation between neurofilament light chain (NFL) in cerebrospinal fluid (CSF) and serum and the relationship between NFL and other biomarkers, subsequent disease activity, and brain volume loss in CIS and RRMS.

**Methods:**

A panel of neurodegenerative and neuroinflammatory markers were analyzed in repeated CSF samples from 41 patients with CIS or RRMS in a prospective longitudinal cohort study and from 22 healthy controls. NFL in serum was analyzed using a single-molecule array (Simoa) method. “No evidence of disease activity-3” (NEDA-3) status and brain volume (brain parenchymal fraction calculated using SyMRI®) were recorded during 4 years of follow-up.

**Results:**

NFL levels in CSF and serum correlated significantly (all samples, *n* = 63, *r* 0.74, *p* < 0.001), but CSF-NFL showed an overall stronger association profile with NEDA-3 status, new T2 lesions, and brain volume loss. CSF-NFL was associated with both new T2 lesions and brain volume loss during follow-up, whereas CSF-CHI3L1 was associated mainly with brain volume loss and CXCL1, CXCL10, CXCL13, CCL22, and MMP-9 were associated mainly with new T2 lesions.

**Conclusions:**

Serum and CSF levels of NFL correlate, but CSF-NFL predicts and reflects disease activity better than S-NFL. CSF-NFL levels are associated with both new T2 lesions and brain volume loss. Our findings further add to the accumulating evidence that CSF-NFL is a clinically useful biomarker in CIS and RRMS and should be considered in the expanding NEDA concept. CSF-CXCL10 and CSF-CSF-CHI3L1 are potential markers of disease activity and brain volume loss, respectively.

**Electronic supplementary material:**

The online version of this article (10.1186/s12974-018-1249-7) contains supplementary material, which is available to authorized users.

## Background

Multiple sclerosis (MS) is a chronic disease characterized by inflammation and degeneration of the central nervous system (CNS). Relapses, new T2 lesions, and disability progression are conventional signs of disease activity in MS. To aid personalized treatment, prognostic biomarkers and biomarkers of disease activity are needed. Assessing brain volume loss and biomarkers in cerebrospinal fluid (CSF) or serum (S) could potentially give a more comprehensive evaluation of disease activity [1–4].

Brain atrophy is present early in the disease course [5, 6] and associated with disability progression [7]. Using SyMRI® to determine brain volume as brain parenchymal fraction (BPF) has been reported as a valid and reproducible method with a clinically acceptable scan time and post-processing time [8]. Neurofilament light chain (NFL) in CSF correlates with long-term prognosis in MS [9], is a risk factor for conversion from clinically isolated syndrome (CIS) to relapsing remitting multiple sclerosis (RRMS) [10], and decreases on treatment with fingolimod and natalizumab [11, 12]. We recently reported that CSF-NFL at baseline predicted disease activity during 2 years of follow-up in patients with CIS and RRMS [13]. S-NFL, determined with sensitive single-molecule array (Simoa) technology [14], has been reported to be highly correlated to CSF levels in RRMS [15].

This study aimed to assess the correlation between S-NFL and CSF-NFL, to evaluate NFL levels in relation to other biomarkers and disease activity parameters and to identify parameters associated with number of new T2 lesions and brain volume loss during 4 years of follow-up in a longitudinal cohort of patients with newly diagnosed CIS and RRMS.

## Methods

### Patients and controls

Forty-one patients with CIS or RRMS were consecutively enrolled in a prospective longitudinal cohort study of CIS and newly diagnosed MS at the Department of Neurology, University Hospital of Linköping, Sweden. All patients fulfilled the revised McDonald criteria from 2010 [16] for CIS or MS. Patients underwent clinical neurological examination including expanded disability status scale (EDSS), blood and CSF sampling as well as MRI at baseline, and at 1, 2, and 4 years of follow-up. Patients received immunomodulatory treatment according to Swedish clinical practice during the study period, from 2009 to 2016. Patient characteristics are presented in Tables 1 and 2. For blood and CSF parameters, 22 age- and sex-matched healthy controls (HC) were recruited from healthy blood donors. Healthy controls were free from past and current neurological and autoimmune disease, and their clinical neurological examinations were normal as were routine findings in CSF (Table 1). No medication, except oral contraceptives, was allowed in healthy controls. The patients and controls were included in a previous study reporting biomarkers in relation to 2 years of follow-up, while in the present study, we report data from 4 years of follow-up and also include BPF and serum NFL.

**Table 1:** Patient and healthy control characteristics at baseline

Clinical and laboratory data	Patients *n* = 41	Healthy controls *n* = 22	*p* value
Women/men (% women)	32/9 (78%)	17/5 (77%)	0.9
Age^a^ (years)	31 (24–36)	32 (26–41)	0.3
Diagnosis (CIS/RRMS)	19/22	N/A	
Relapse within last 2 months (yes/no)	23/18	N/A	
Mean disease duration^b^ (months)	11.8	N/A	
Median disease duration^b^ (months)	3.5	N/A	
Disease duration^b^ (number of subjects)		N/A	
0–1 months	*10*		
1.25–2 months	*7*		
2.25–3 months	*3*		
3.25–6 months	*6*		
6.5–12 months	*7*		
13–24 months	*3*		
25–36 months	*2*		
37–48 months	*1*		
49–120 months	*2*		
Median EDSS	2.0	N/A	
EDSS (number of subjects)			
0	*6*	*22*	
1.0	*12*		
1.5	*2*		
2.0	*12*		
2.5	*3*		
3.0	*1*		
3.5	*2*		
4.0	*2*		
4.5	*1*		
CSF mononuclear cell count^a^	4.6 × 10^6^/L (1.8–9.2)	2.1 × 10^6^/L (0.9–2.7)	0.001
Albumin ratio^a^	4.8 (3.4–6.0)	4.7 (3.6–5.3)	0.5
IgG index^a^	0.7 (0.5–1.1)	0.5 (0.5–0.5)	< 0.001
IgG synthesis index^a^	1.3 (1.0–2.1)	0.9 (0.9–1.0)	< 0.001
Oligoclonal CSF IgG bands (pos/neg)	33/8	0/22	< 0.001

*p* values from chi-square test for sex distribution and oligoclonal bands and from Mann-Whitney *U* test for age and CSF data

*N/A* not applicable, *DMT* disease-modifying treatment

^a^Median and within brackets interquartile range

^b^Disease duration refers to time from first symptom suggestive of demyelinating disease

**Table 2:** Patient diagnoses, relapse status, and treatment status over time

Clinical and laboratory data	Baseline	1 year	2 years	4 years
Number of subjects	41	41	40	39
Diagnosis (CIS/RRMS)	19/22	12/29	9/31	7/32
Relapse within last 2 months (yes/no)	23/18	4/37	2/38	2/37
Treatment (number of subjects)				
No DMT	41	17	18	18
Interferon-β 1b	0	18	12	5
Interferon-β 1a	0	1	1	1
Dimetylfumarate	0	0	0	2
Fingolimod	0	1	2	3
Natalizumab	0	4	7	10

### Disease activity assessment

All clinical assessments regarding relapses and EDSS were performed by the same neurologist (IH), and all MRI examinations were thoroughly reviewed by the same neuroradiologist. Patients that showed no relapses, no brain MRI activity (no new or enlarging T2 lesions or Gadolinium-enhancing lesions), and no sustained disability worsening (EDSS progression) during follow-up were classified as showing “no evidence of disease activity”-3 (NEDA-3) (*n* = 20 at 1 year, *n* = 12 at 2 years, and *n* = 7 at 4 years), while patients with relapses, brain MRI activity, or sustained disability worsening were classified as showing evidence of disease activity (EDA-3) (*n* = 21 at 1 year, *n* = 27 at 2 years, and *n* = 32 at 4 years). All 41 patients were evaluated at 1 year and 39 patients were evaluated at 2 and 4 years of follow-up. At 2 years, one pregnant patient did not undergo MRI and one patient had left the study. At 4 years, one additional patient had left the study, while the previously pregnant patient completed 4-year follow-up.

### Cerebrospinal fluid and serum analyses

All CSF sampling was carried out by the same neurologist (IH), and CSF was always collected 8–12 a.m. Serum samples were collected directly after CSF collection. One aliquot of the CSF sample was used for cell counting, CSF/serum albumin ratio, IgG index, IgG synthesis index, and isoelectric focusing, all according to clinical routines performed at the Department of Clinical Chemistry, Linköping University Hospital. Within 1 hour, the remaining CSF was centrifuged (300×*g* for 10 min.) and the supernatant was aliquoted and immediately frozen and stored at − 70 °C.

CSF samples were analyzed for chemokine concentrations with a multiplex bead assay (Milliplex® MAP kits, EMD Millipore Corporation, St. Charles, MO, USA) according to the manufacturer’s instructions, except that an additional lower standard point was added to the standard curve. The measurements were performed using Luminex®200™ (Invitrogen, Merelbeke, Belgium). For data acquisition, the software program xPONENT 3.1™ (Luminex Corporation, Austin, TX, USA) was used, and for data analysis, MasterPlex® Reader Fit was used. The detection limits were 16 pg/mL for CXCL1, CXCL10, and CCL22; 3.2 pg/mL for CXCL8; and 3.9 pg/mL for CXCL13. Values below the detection limit were assigned half the value of the detection limit.

CSF NFL concentration was measured using the NF-light assay according to instructions from the manufacturer (UmanDiagnostics, Umeå, Sweden). CSF NFH concentration was measured using the Phosphorylated NEFH (Human) ELISA Kit according to instructions from the manufacturer (Abnova, Taipei City, Taiwan). CSF MMP-9 concentration was measured using the Human MMP-9 Base Kit according to instructions from the manufacturer (Meso Scale Discovery, Rockville, MD). CSF GFAP concentration was measured using an in-house ELISA as previously described [17]. CSF CHI3L1 and OPN concentrations were measured using commercially available ELISAs (R&D Systems, Inc. Minneapolis, MN). The lower limits of quantification for the NFH and MMP-9 assays were 31.2 and 122 pg/mL, respectively. For the other analytes, all samples had concentrations within the quantifiable range of the assay. All measurements were performed in one round of experiments using one batch of reagents by board-certified laboratory technicians who were blinded to clinical information. Intra-assay coefficients of variation were below 15%.

S-NFL concentration was measured using the NF-Light kit from UmanDiagnostics (UmanDiagnostics, Umeå, Sweden), transferred onto the Simoa platform using a homebrew kit (Quanterix Corp, Boston, MA, USA), as previously described in detail [18]. The lower limit of quantification (LLoQ), determined by the blank mean signal + 10 SD, was 1.95 pg/mL. Levels in all samples were well above LLoQ. The analyses were performed by board-certified laboratory technicians using one batch of reagents with intra- and inter-assay coefficients of variation below 10 and 15%, respectively.

### Magnetic resonance imaging and post processing

All subjects were examined on a 1.5-T Philips Achieve MRI scanner (Philips Healthcare, Best, The Netherlands) using an eight-channel phased array head coil. Quantitative MRI images were acquired using QMAP sequence [19]. BPF was calculated using SyMRI® version 8.0 (SyntheticMR, Linköping, Sweden).

### Statistical analyses

Statistical analyses were performed using SPSS for Windows, version 23. Analyzing data sets with non-Gaussian distribution, the Mann-Whitney *U* test was used to compare two groups and non-parametric bivariate correlation analysis (Spearman) was used for correlation analyses. The relationship between NFL in CSF and serum was examined using bivariate linear regression and Spearman correlation analysis, as well as Pearson correlation analysis when sample size was > 50. Friedman’s test with Dunn correction for multiple comparisons was used to compare repeated measurements of immunological markers in patients over time. Repeated measures ANOVA with Bonferroni correction for multiple comparisons was used to compare repeated BPF measurements in patients over time. Multiple linear regression analysis was used to evaluate brain volume loss over time and number of new T2 lesions over time. Logistic regression was used when investigating NFL and other markers in relation to NEDA. Receiver operating characteristic (ROC) curves were derived from logistic regression to investigate the discriminatory power of NFL and other markers between patients with and without disease activity during follow-up. Because of multiple testing, a stringent *p* value of ≤ 0.01 was considered to be significant in Mann-Whitney *U* tests, *t* tests, repeated measures ANOVA, and linear regression analyses. In Spearman correlation analyses, a very stringent significance level of Spearman’s rho ≥ 0.5 and *p* ≤ 0.01 was used, which entailed a maximum type II error of 0.21 when *n* = 41 and 0.27 when *n* = 37. For comparisons within the CIS group, where patient numbers were small and comparisons few, a *p* value of ≤ 0.05 was considered significant in Mann-Whitney *U* tests. Area under curve (AUC) values were compared using the DeLong method, in MEDCALC®, with the significance level set at 0.05. All *p* values were based on two-tailed statistical tests.

## Results

### S-NFL and CSF-NFL correlate, and both are elevated in patients at baseline

**Fig. 1:**
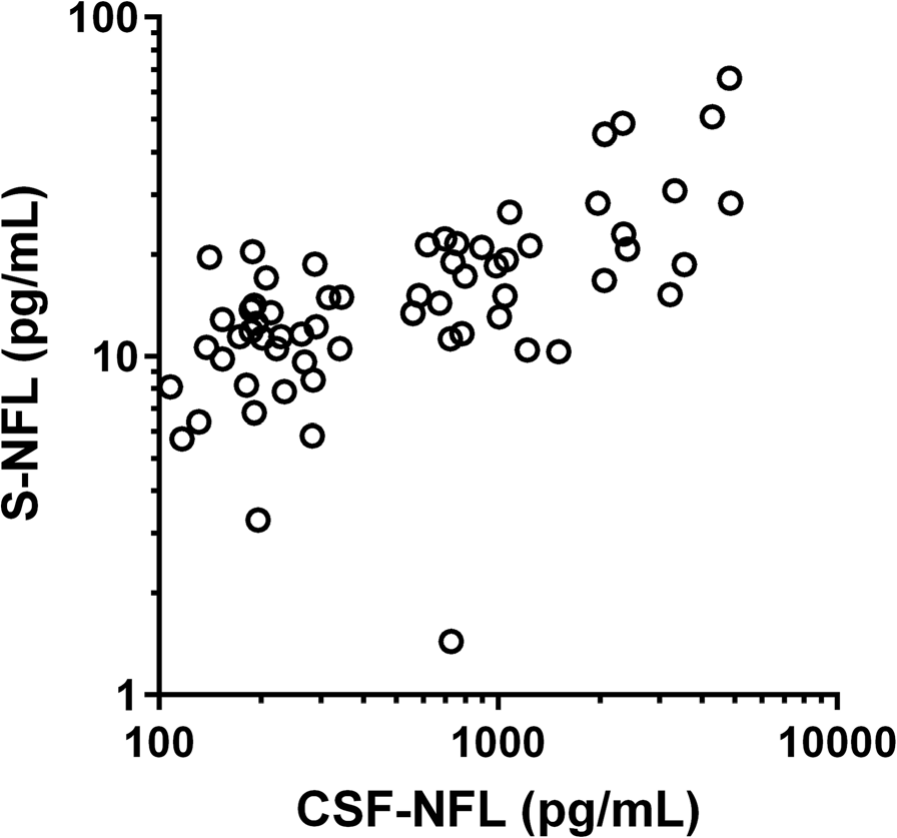
Correlation between S-NFL and CSF-NFL. Correlation between S-NFL and CSF-NFL, all samples from patients and healthy controls (*n* = 63). Logarithmic scales are used on graph axes for improved visibility (increased separation of data points), whereas statistical analyses were performed on non-transformed linear data. Spearman’s rho 0.65 (*p* < 0.001) and Pearson’s *r* 0.74 (*p* < 0.001)

S-NFL and CSF-NFL correlated in HC (*n* = 22, Spearman’s rho 0.59, *p* = 0.004), in patients at baseline (*n* = 41, Spearman’s rho 0.63, *p* < 0.001), in samples from patients at baseline and HC combined (*n* = 63, Spearman’s rho 0.65, *p* < 0.001, and Pearson’s *r* 0.74 (*p* < 0.001). *R*^2^ from bivariate linear regression with S-NFL as dependent variable and CSF-NFL as independent variable (*n* = 63) was 0.54, *p* < 0.001, equation y = 0.007*x* + 10.1. A correlation plot is presented in Fig. 1.

**Fig. 2:**
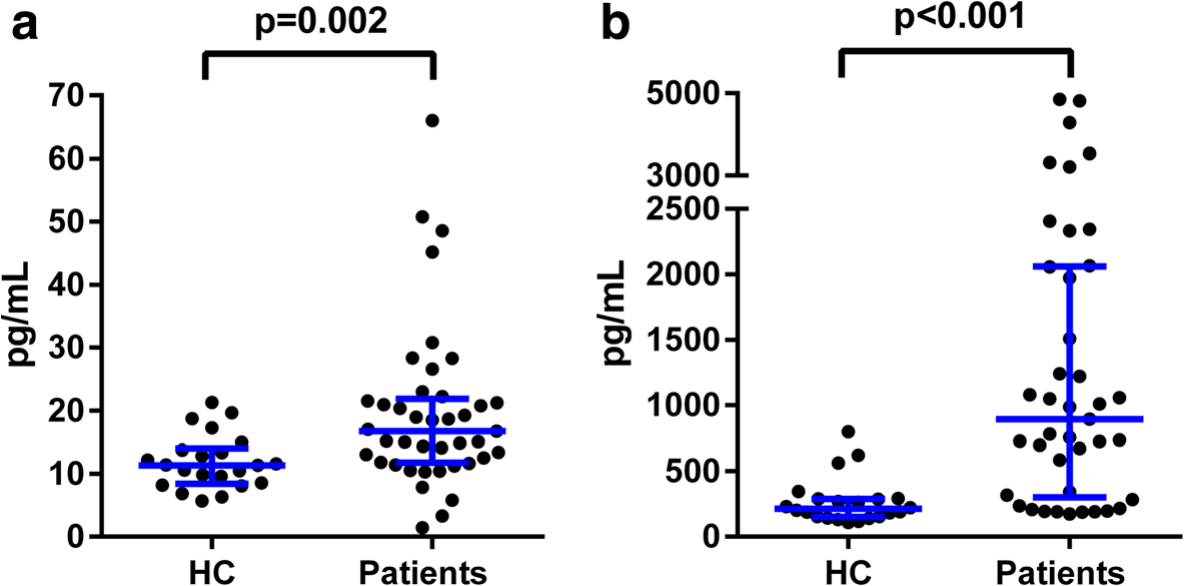
NFL in serum (**a**) and CSF (**b**) in healthy controls and patients at baseline. Lines show median and interquartile range. For S-NFL, patient group (*n* = 41) median was 17 pg/mL (interquartile range 12–22 pg/mL) and healthy control (HC) (*n* = 22) group median was 11 pg/mL (interquartile range 8–14 pg/mL). For CSF-NFL, patient group median was 895 pg/mL (interquartile range 300–2060 pg/mL) and HC group median was 212 pg/mL (interquartile range 151–289 pg/mL). *P*- values from Mann-Whitney *U* test

To compare NFL levels in health and disease, patients at baseline (all untreated) were compared with healthy controls. Both S-NFL and CSF-NFL were significantly higher in patients than in controls, see Fig. 2.

In patients, neither CSF nor serum NFL could be explained by age (*R*^2^ 0.01, *p* = 0.5 for CSF-NFL and *R*^2^ 0.003, *p* = 0.7 for S-NFL in simple linear regression). In healthy controls, on the other hand, age was a significant factor in relation to both CSF-NFL and S-NFL levels (*R*^2^ 0.73, *p* < 0.001 for CSF-NFL and *R*^2^ 0.30, *p* = 0.008 for S-NFL in simple linear regression). S-NFL levels, just like recently reported for CSF-NFL levels [13] in this cohort, did not differ between CIS patients and RRMS patients (*p* = 0.9 for S-NFL and *p* = 0.4 for CSF-NFL) or between patients with and without a relapse starting within 2 months prior to sample collection at baseline (*p* = 0.7 for S-NFL and *p* = 0.1 for CSF-NFL). However, when including not only baseline samples but also follow-up samples, thereby increasing total *n* from 41 to 161, RRMS patients had higher CSF-NFL (*p* = 0.01), but not S-NFL (*p* = 0.6), than CIS patients, and NFL levels were higher in relapse patients than in non-relapse patients (*p* = 0.004 for S-NFL and *p* ≤ 0.001 for CSF-NFL).

### Neuroinflammatory and neurodegenerative markers at baseline and over time

CSF and serum was collected at baseline and after 1, 2, and 4 years. The complete descriptive data are presented in an additional table (see Additional file 1: Table S1) for neuroinflammatory and neurodegenerative markers (NFL, neurofilament heavy chain (NFH), glial fibrillary acidic protein (GFAP), chitinase 3 like 1 (CHI3L1), matrix metalloproteinase 9 (MMP-9), osteopontin (OPN), CXCL1, CXCL8, CXCL10, CXCL13, and CCL22). Data on levels in healthy controls and in patients at baseline have been reported previously [13].

#### NFL levels at baseline, in particular in CSF, predict NEDA-3 status during follow-up

**Fig. 3:**
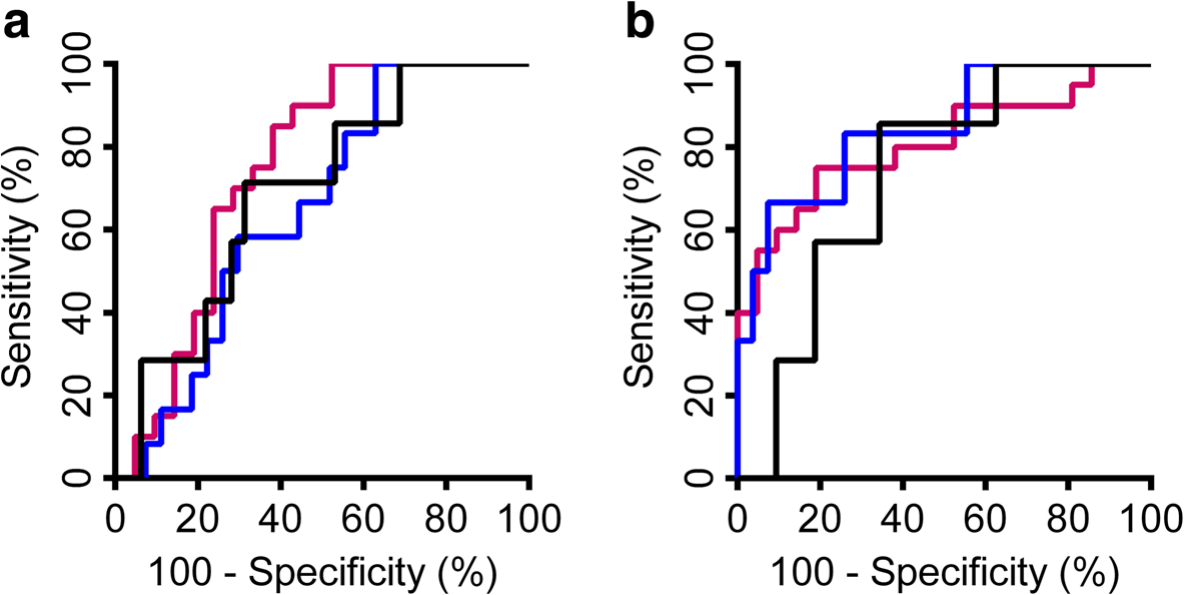
S-NFL and CSF-NFL and separation of patients with and without disease activity during follow-up. Receiver operating characteristics (ROC) curves for baseline **a** S-NFL and **b** CSF-NFL and separation of patients with and without disease activity during follow-up after 1 (purple), 2 (blue), and 4 (black) years. Disease activity was defined as relapses, brain MRI activity (new or enlarging T2 lesions or Gd-enhancing lesions), or disability worsening. With a cutoff level of 450 ng/L for baseline CSF-NFL, sensitivity for identifying disease activity was 95% at 1 year, 93% at 2 years, and 81% at 4 years, with specificity 50, 67, and 57%, respectively. With a cutoff level of 14.2 ng/L for baseline S-NFL, sensitivity for identifying disease activity was 76% at 1 year, 74% at 2 years, and 72% at 4 years, with specificity 50, 50, and 57%, respectively

Area under curve (AUC) for baseline levels of NFL in relation to disease activity during 1, 2, and 4 years of follow-up were 0.75, 0.65, and 0.69 for S-NFL, and 0.81, 0.85, and 0.73 for CSF-NFL; ROC curves are presented in Fig. 3. The AUC value was significantly higher at 2 years for CSF-NFL than S-NFL (*p* = 0.05), but did not differ significantly at 1 and 4 years (*p* > 0.05). With a cutoff value of ≥ 450 ng/L for baseline CSF-NFL, the sensitivity of identifying patients with disease activity was 95% at 1 year, 93% at 2 years, and 81% at 4 years, with specificity 50, 67, and 57%, respectively. With a cutoff value of 14.2 ng/L for baseline S-NFL, sensitivity of identifying patients with disease activity was 76% at 1 year, 74% at 2 years, and 72% at 4 years, with specificity 50, 50, and 57%, respectively. Thus, low levels of CSF-NFL entail a high probability of NEDA during follow-up.

#### Chemokines at baseline predict NEDA-3 status during follow-up

For prediction of NEDA-3/EDA-3 during 1 and 2 years of follow-up, baseline CXCL10 and baseline CSF-NFL had the highest ROC-based AUCs (0.81–0.85) and for prediction of NEDA-3/EDA-3 during 4 years of follow-up, baseline MMP-9, CXCL10, CCL22, and CXCL13 had the highest AUCs (0.80–0.83), see Additional file 2: Table S2 for predictive values (AUCs) of the whole panel of markers. There were no statistically significant differences between AUCs ≥ 0.80 (all *p* ≥ 0.05). CXCL10 was the only marker with all three (1, 2, and 4 years) AUCs above 0.8.

#### NFL levels during follow-up are associated with NEDA-3 status during follow-up

When evaluating associations with disease activity, both in the cross-sectional and the longitudinal perspective, AUCs from follow-up levels as well as mean levels of follow-up levels were assessed, in addition to AUCs from baseline levels (table presented in Additional file 2: Table S2). The marker with the highest AUC for NEDA-3/EDA-3 during 1 year of follow-up was CSF-NFL at 1 year (AUC 0.89), and for 2 years, it was mean CSF-NFL during 2 years of follow-up (AUC 0.88). Regarding NEDA-3/EDA-3 during 4 years of follow-up, the highest NFL AUC (mean CSF-NFL during 4 years of follow-up, AUC 0.76) was surpassed by others in absolute numbers (see Additional file 2: Table S2), although not significantly different (*p* ≥ 0.05). These data show that NFL levels in CSF during follow-up, especially mean levels, are highly associated with ongoing disease activity expressed as NEDA-3/EDA-3.

### Brain parenchymal fraction decreases over time and is associated with increased disease activity in the early phase

**Fig. 4:**
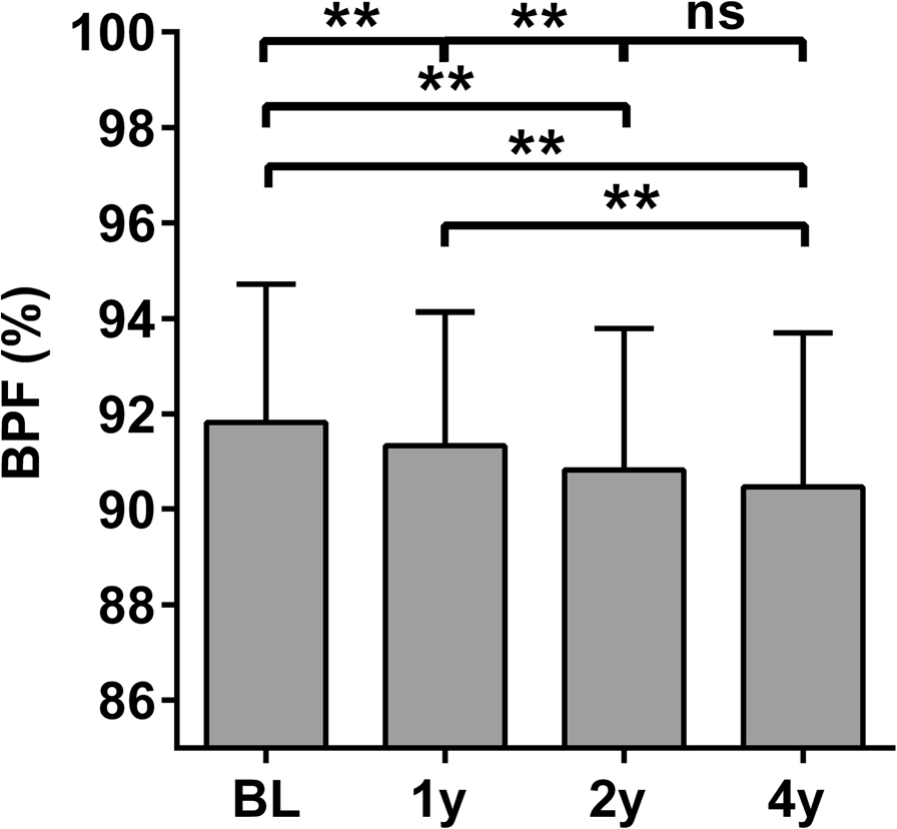
Brain parenchymal fraction in patients at baseline and during follow-up. Brain parenchymal fraction (BPF) in patients at baseline (BL) and at 1 year (1y), 2 years (2y), and 4 years (4y) of follow-up. **p ≤ 0.01 in repeated measures ANOVA with Bonferroni correction for multiple comparisons. ns: not significant

One patient was excluded at all four time points due to very low BPF associated with congenital hydrocephalus. Three patients were excluded due to missing data. BPF data from the 37 patients that completed all four BPF measurements are presented in Fig. 4. BPF mean ± SD was 91.8 ± 2.9 at baseline, 91.3 ± 2.8 at 1 year follow-up, 90.8 ± 3.0 at 2 years follow-up, and 90.5 ± 3.2 at 4 years follow-up. In repeated measures ANOVA with Bonferroni correction for multiple comparisons, BPF at 1, 2, and 4 years of follow-up were significantly lower than at baseline (*p* < 0.005, *p* < 0.001, and *p* < 0.001) and BPF at 2 and 4 years were significantly lower than at 1 year (*p* < 0.001 and 0.004). However, BPF at 4 years was not lower than at 2 years (*p* = 0.9). Time from baseline MRI to follow-up MRI (mean ± SD) was 12 ± 1 months, 24 ± 1 months, and 44 ± 5 months for 1-, 2-, and 4-year follow-up, respectively.

Mean BPF decrease was 0.49/year from baseline to 1 year, 0.50/year from baseline to 2 years, and 0.36/year from baseline to 4 years. Mean BPF decrease from baseline to 4-year follow-up was significantly higher in patients with EDA-3 than in patients with NEDA-3 during 1 year (− 1.6 vs − 0.7, *p* = 0.001), but not two (− 1.4 vs − 0.7, *p* = 0.02) and four (− 1.3 vs − 0.6, *p* = 0.06) years of follow-up.

### Neurofilament levels correlate with both new T2 lesions and BPF decrease, while chemokines correlate with new T2 lesions and CHI3L1 correlates with BPF decrease

Correlations between the panel of biomarkers (listed in Additional file 1: Table S1) and baseline BPF, BPF decrease over time, baseline number of T2 lesions in brain MRI, and number of new T2 lesions over time were examined in 37 patients with complete data sets of BPF and the other parameters. Table 3 shows a map of the significant correlations. Of note, neurofilament levels correlated with both number of new T2 lesions and BPF decrease during follow-up, whereas inflammatory chemokines correlated only with new T2 lesions and CHI3L1 levels correlated only with BPF decrease.

**Table 3:** Correlations between neuroinflammatory and neurodegenerative markers, baseline number of T2 lesions, number of new T2 lesions, and BPF change during follow-up

	T2, BL	New T2, 1 year	New T2, 2 years	New T2, 4 years	BPF change,4 years
S-NFL, BL	0.33	0.55*	0.43	0.44	− 0.24
S-NFL, 1y	0.45	0.43	0.33	0.34	− 0.56*
S-NFL, 2y mean	0.45	0.52*	0.37	0.37	− 0.37
S-NFL, 4y mean	0.41	0.43	0.32	0.35	−0.42
CSF-NFL, BL	0.29	0.60*	0.64*	0.51*	− 0.20
CSF-NFL, 1y	0.59*	0.59*	0.58*	0.50*	− 0.54*
CSF-NFL, 2y mean	0.43	0.62*	0.65	0.54	−0.33
CSF-NFL, 4y mean	0.41	0.55*	0.60*	0.53*	− 0.35
MMP-9, BL	0.46	0.53*	0.47	0.42	− 0.16
MMP-9, mean	0.38	0.51*	0.51*	0.46	−0.26
CHI3L1, BL	0.49	0.42	0.36	0.26	−0.55*
CHI3L1, 1y	0.45	0.35	0.30	0.30	− 0.60*
CHI3L1, 2y	0.31	0.13	0.11	0.13	− 0.50*
CHI3L1, mean	0.42	0.30	0.24	0.20	− 0.54*
OPN, BL	0.51*	0.34	0.21	0.23	− 0.52*
CXCL1, BL	0.51*	0.56*	0.42	0.23	− 0.34
CXCL10, BL	0.50*	0.58*	0.48	0.41	− 0.26
CXCL10, mean	0.47	0.51*	0.42	0.34	− 0.37
CXCL13, BL	0.37	0.51*	0.52*	0.46	− 0.26
CXCL13, mean	0.40	0.49	0.52*	0.45	− 0.33
CCL22, mean	0.29	0.49	0.57*	0.54*	− 0.31
T2, BL	1.00	0.48	0.39	0.27	− 0.56*
New T2, 1y	0.48	1.00	0.86*	0.66*	− 0.44
New T2, 2y	0.39	0.86*	1.00	0.83*	− 0.47
New T2, 4y	0.27	0.66*	0.83*	1.00	− 0.36
BPF decrease, 4y	− 0.56*	− 0.44	− 0.47	− 0.36	1.00

Magnitude of correlation coefficients (Spearman’s rho) from bivariate non-parametric correlation analyses (Spearman) are shown

2y mean refers to the mean of levels at baseline, 1-year, and 2-year follow-up

4y mean refers to the mean of levels at baseline, 1-year, 2-year, and 4-year follow-up

T2, BL: number of T2 lesions in baseline brain MRI

New T2, 1y, 2y and 4y: number of new T2 lesions in brain MRI during 1, 2, and 4 years of follow-up, respectively, compared to baseline MRI

BPF change, 4y: brain parenchymal fraction decrease during 4 years of follow-up

*S* serum, *CSF* cerebrospinal fluid, *BL* baseline

*Spearman’s rho ≥ 0.50 and *p* ≤ 0.001

### Linear regression modeling indicates CHI3L1 as the strongest predictor of BPF decrease

To identify the most important parameters for prediction (using baseline parameters only) of, and for association (follow-up data included as well) with, BPF decrease and new T2 lesions, linear regression modeling of BPF decrease and new T2 lesions during 4 years of follow-up was performed. Details about these procedures are given in Supplemental data. For prediction of BPF decrease, baseline CHI3L1 was the single parameter that performed best (*R*^2^ 0.26, *p* = 0.001) and a peak adjusted *R*^2^ of 0.41 (*p* = 0.001) was noted for a model including age, CSF-mononuclear cells, NFH, MMP-9, CHI3L1, and T2 lesions at baseline. As for association with BPF decrease, adjusted *R*^2^ peaked with a model including baseline T2 lesions, baseline OPN, and 1-year CHI3L1 (adjusted *R*^2^ 0.30, *p* = 0.002), and the single parameter that performed best was 1-year CHI3L1 (*R*^2^ 0.29, *p* = 0.001). For prediction of new T2 lesions, no model reached the significance level *p* ≤ 0.01, whereas for association with new T2 lesions, adjusted *R*^2^ peaked with a model including mean CCL22, 1-year CSF-NFL, and baseline CXCL13 (adjusted *R*^2^ 0.40, *p* < 0.001), and the single parameter that performed best was 1-year CSF-NFL (*R*^2^ 0.32, *p* < 0.001). Details of linear regression statistics are given in Additional file 3.

### CIS converters

Within the CIS group at baseline (*n* = 19), CXCL10, NFL, and MMP-9 in CSF were significantly higher (*p* ≤ 0.05 in Mann-Whitney *U* tests) in patients that converted to RRMS during 4 years of follow-up (*n* = 12) than in non-converters (*n* = 7), whereas S-NFL (*p* = 0.10) and the other markers (*p* = 0.07–0.90) did not differ. For the 12 CIS patients that converted to RRMS during the study, multiple regression modeling of time to conversion resulted in a highly significant model (adjusted *R*^2^ 0.81, *p* = 0.006) when including baseline CSF levels of CXCL1, CXCL10, CXCL13, and NFL, and number of T2 lesions at baseline).

## Discussion

In the present study, we report a correlation between NFL levels in CSF and serum (Pearson’s *r* 0.74) that is in line with what has been reported by others [15, 20]. Although baseline S-NFL was correlated with subsequent disease activity, CSF NFL seems to reflect disease activity better since baseline CSF-NFL predicted NEDA-3 during 2 years of follow-up significantly better than S-NFL, and moreover, CSF-NFL levels showed an overall stronger correlation profile than serum levels with regard to number of new T2 lesions. Our findings confirm CSF-NFL as a prognostic biomarker for conversion from CIS to MS [13, 21], association with disease activity [9, 13], and change in brain volume [22]. The fact that all these findings were significant in our moderately sized cohort, and the finding that CSF-NFL performed well compared with other markers and showed associations with both disease activity and brain volume loss, render further support for CSF-NFL as a clinically useful biomarker in MS.

Interestingly, 1-year CSF-NFL levels correlated significantly with both new T2 lesions and BPF decrease during 4 years of follow-up. For other markers, they were either associated with BPF decrease (CHI3L1 and OPN) or with new T2 lesions (CXCL1, CXCL10, CXCL13, CCL22, and MMP-9). In linear regression modeling of new T2 lesions, the combination of 1-year CSF-NFL, CCL22 (mean level) and CXCL13 (baseline level) performed better than 1-year CSF-NFL alone, suggesting an added value of combining several markers, which was recently shown for chemokines and OPN [23]. Of note, chemokines, in contrast to most cytokines, are present at measurable levels in CSF. We here showed that CXCL10 was able to predict disease activity at all follow-up time points (NEDA-3 status at 1, 2, and 4 years) and that CXCL10 was also higher in CIS-converters than in non-converters, findings that suggest CXCL10 as a biomarker in CIS and RRMS. This has been indicated in other studies as well [24, 25]. As for brain volume loss, CHI3L1 had the highest predictive value, and further studies may confirm its clinical role.

Expanding NEDA-3 to NEDA-4 and NEDA-5 by including brain volume loss and biomarkers could improve assessment of disease activity in MS. Number of T2 lesions and T2 lesion volume in conventional MRI correlate poorly to clinical disease manifestations and disease progression in MS [26], although lesion volume has been reported to predict long-term disability [27]. Measuring brain volume loss as BPF decrease is non-invasive and logistically easy, only marginally extending time in the MRI-scanner. However, the use of BPF measurements and other brain volume quantification methods are hampered by technical limitations, physiological variation, and difficulties in identifying optimal cutoff levels for annual brain volume loss [3, 28]. It has been argued that although suitable for cohort studies, BPF measurements do not seem adequate for assessing changes in individual patients over months or a few years [28]. CSF-NFL, on the other hand, is a biomarker that provides clinically useful information and therefore emerges as a strong candidate for inclusion in the NEDA concept, although cutoff levels need to be settled. As for the more feasible S-NFL, a reasonable correlation with CSF-NFL and an ability to reflect disease activity in MS [15, 29, 30] was here confirmed, reiterating the suggestion of S-NFL as a promising biomarker. Indeed, recently published data from a large MS cohort show that higher S-NFL is clearly associated with worse clinical (EDSS progression) as well as MRI (T2 lesions and atrophy) outcome [31]. CSF-NFL was however not reported, and in our cohort, S-NFL did not perform as well as in that study [31]. It could be that smaller sample size affected our results, but still we noted that CSF-NFL yielded strong results in our single-center cohort. The exciting development of reliable high sensitivity assays has enabled detection of brain-derived markers in serum and plasma, but still these levels constitute a proxy for the levels in CSF. As long as there is no absolute correlation between CSF- and S-NFL, it seems clear that CSF levels should better reflect CNS pathology than blood, as supported by the stronger associations that we see between CSF-NFL and NEDA-3 as well as T2 lesions and brain volume, compared with S-NFL. Also, some markers of inflammation, such as the chemokines CXCL10 and CXCL13, have been reported to be elevated and to correlate with disease activity when measured in the CSF, but not in plasma [13, 32, 33]. Since CSF analysis is recommended in the diagnostic process [34], it is uncontroversial and appropriate to include NFL and other biomarkers in CSF at this stage. Also in monitoring of disease activity and treatment effects, the best possible medical information would be obtained by CSF analyses, which in the future also may include tools to personalized treatment [23, 35]. On the other hand, aspects like patient discomfort and various practical issues limit the use of CSF biomarkers, whereas blood samples can usually be easily obtained. We believe that both CSF- and S-NFL should be part of the initial diagnostic work-up for all patients and that follow-up CSF analyses should be considered when as complete as possible information is required to make a clinical decision. For many patients follow-up CSF analyses may not be motivated, whereas S-NFL could be liberally considered as an addition to NEDA-3 evaluation. As for the other neuroinflammatory and neurodegenerative markers in our panel, we have previously reported that chemokine levels in plasma did not differ significantly between patients and healthy controls [13]. There have been reports by others that e.g. CHI3L1 in serum may be a useful biomarker [36, 37], but so far, S-NFL shows most promise [31].

Limitations of this study include the cohort size, varying treatment and lack of MRI data from healthy controls. Varying treatment regimens in the cohort could lower the prognostic value of baseline biomarker levels. However, despite the fact that some patients received disease modifying drugs during follow-up, baseline levels of some proteins clearly showed prognostic value. We also focused on NEDA status, new T2 lesions, and BPF decrease in relation to follow-up levels and mean levels of NFL, etc.*,* thereby exploring association over time instead of prediction. When examining, for example, the association between mean level of NFL during follow-up and NEDA-3 status during follow-up, the impact of treatment is negligible or small unless treatment affect NFL and NEDA-3 status very unevenly. Study strengths are the control group, consisting of sex- and age-matched healthy individuals, that the patient group consisted of well-characterized CIS and MS patients examined and thoroughly followed up prospectively by the same neurologist in a standardized way and that all patients were untreated at baseline. Also, a broad panel of potential biomarkers was included. The present study provides 4-year follow-up data on CSF-NFL in a patient cohort that we have previously presented 2-year follow-up data from, and in addition, we report S-NFL data as well as brain volume analyses for the entire study period (4 years). Taken together, this is the first study that reports longitudinal data on these markers, including NFL levels in CSF and serum, in relation to both NEDA-3 and brain volume loss in a clinical setting study.

## Conclusions

We show that although serum and CSF levels of NFL correlate significantly, CSF-NFL predicts and reflects disease activity better than S-NFL during follow-up in our cohort of patients with CIS and RRMS. CSF-NFL is associated with both new T2 lesions and brain volume loss during follow-up. Our findings further add to the accumulating evidence that CSF-NFL is a useful biomarker in CIS and RRMS and should be considered in the expanding NEDA concept. Our findings also support S-NFL as a potential biomarker in CIS and RRMS. Other biomarkers that need further attention include CXCL10 and CHI3L1 for prediction of NEDA-3 and brain volume loss, respectively.

## Additional files

Additional file 1: **Table S1.** Neurodegenerative and neuroinflammatory markers in CSF (and for NFL also in serum) over time in patients with CIS and RRMS. (DOCX 23 kb)

Additional file 2: **Table S2.** Neuroinflammatory and neurodegenerative markers with respect to separation of patients with and without disease activity during follow-up. (DOCX 17 kb)

Additional file 3: Linear regression modeling details. (DOCX 12 kb)

## Abbreviations

AUC: Area under curve
BPF: Brain parenchymal fraction
CHI3L1: Chitinase 3 like 1
CIS: Clinically isolated syndrome
CNS: Central nervous system
CSF: Cerebrospinal fluid
EDA: Evidence of disease activity
EDSS: Expanded disability status scale
GFAP: Glial fibrillary acidic protein
HC: Healthy controls
MMP-9: Matrix metalloproteinase 9
MS: Multiple sclerosis
NEDA: No evidence of disease activity
NFH: Neurofilament heavy chain
NFL: Neurofilament light chain
OPN: Osteopontin
ROC: Receiver operating characteristic
RRMS: Relapsing remitting multiple sclerosis
S: Serum
