# Supplementary material for: Neurofilament levels, disease activity and brain volume during follow-up in multiple sclerosis
Source: J Neuroinflammation. 2018 Jul 18;15:209. doi: 10.1186/s12974-018-1249-7 (PMC6052680; doi:10.1186/s12974-018-1249-7)
Supplement: Supplementary file 1 — Table S1. Neurodegenerative and neuroinflammatory markers in CSF (and for NFL also in serum) over time in patients with CIS and RRMS. (DOCX 23 kb) [file 12974_2018_1249_MOESM1_ESM.docx]

**Table S1.** Neurodegenerative and neuroinflammatory markers in CSF (and for NFL also in serum) over time in patients with CIS and RRMS

|  | **Baseline**  **n=41** | **One year**  **n=41** | **Two years**  **n=40** | **Four years**  **n=39** | **Measurable levels,**  **% of patients** |
| --- | --- | --- | --- | --- | --- |
| CXCL1 | 17 (8-23) | 17 (8-26) | 16 (8-23) | N/A | 59, 56, 55 |
| CXCL8 | 25 (22-30) | 26 (21-34) | 25 (19-32) | N/A | 100, 100, 100 |
| CXCL10 | 1091 (814-2093) | 1404 (744-1861) | 1286 (710-1796) | N/A | 100, 100, 100 |
| CXCL13 | 4 (2-17) | 2 (2-14) | 2 (2-7) | N/A | 56, 44, 32 |
| CCL22 | 21 (8-57) | 8 (8-29) | 8 (8-27)***** | N/A | 68, 49, 42 |
| Serum NFL | 17 (12-22) | 14 (8-20)***** | 13 (9-16)***** | 11 (8-16)***** | 100, 100, 100, 100 |
| NFL | 895 (300-2061) | 381 (248-682)***** | 327 (202-513)***** | 374 (242-515)***** | 100, 100, 100, 100 |
| NFH | 35 (16-67) | 32 (16-51) | 16 (16-35)***** | N/A | 61, 51, 37 |
| MMP-9 | 221 (61-625) | 130 (61-327)***** | 61 (61-180)***** | N/A | 68, 54, 37 |
| GFAP | 464 (348-636) | 497 (371-626) | 471 (364-570) | N/A | 100, 100, 100 |
| CHI3L1 | 96362  (75541-177092) | 97724  (72779-152230) | 89830  (71078-150205) | N/A | 100, 100, 100 |
| OPN | 83 (52-125) | 79 (54-119) | 84 (56-122) | N/A | 100, 100, 100 |
| Data on concentrations in cerebrospinal fluid are presented. For NFL, serum levels are also presented.  Concentrations are given in pg/mL and presented as median and within brackets interquartile range.  Measurable levels column states the percentage of patients with measurable levels at baseline and at consecutive follow-ups. Baseline levels in patients have been reported previously[1]. The follow-up levels that we now report should be viewed in the context of heterogeneity in treatment and potential treatment effect during follow-up.  ***** Significantly lower level at follow-up than at baseline, p-values from Friedman test followed by Dunn´s test to correct for multiple comparisons ≤ 0.01. N/A: not available | | | | | |

**REFERENCE**

1. Hakansson I, Tisell A, Cassel P, Blennow K, Zetterberg H, Lundberg P, Dahle C, Vrethem M, Ernerudh J: **Neurofilament light chain in cerebrospinal fluid and prediction of disease activity in clinically isolated syndrome and relapsing-remitting multiple sclerosis.** *Eur J Neurol* 2017, **24:**703-712.
