# Supplementary material for: Neurofilament levels, disease activity and brain volume during follow-up in multiple sclerosis
Source: J Neuroinflammation. 2018 Jul 18;15:209. doi: 10.1186/s12974-018-1249-7 (PMC6052680; doi:10.1186/s12974-018-1249-7)
Supplement: Supplementary file 2 — Table S2. Neuroinflammatory and neurodegenerative markers with respect to separation of patients with and without disease activity during follow-up. (DOCX 17 kb) [file 12974_2018_1249_MOESM2_ESM.docx]

**Table S2.** Neuroinflammatory and neurodegenerative markers with respect to separation of patients with and without disease activity during follow-up

|  | **1y AUC**  **EDA-3/NEDA-3** | **2y AUC**  **EDA-3/NEDA-3** | **4y AUC**  **EDA-3/NEDA-3** |
| --- | --- | --- | --- |
| S NFL, BL | 0.75 | 0.65 | 0.69 |
| S NFL, 1y | 0.74 | 0.64 | 0.48 |
| S NFL, 2y | 0.63 | 0.51 | 0.41 |
| S NFL, 4y | 0.56 | 0.47 | 0.45 |
| S NFL, 2y mean | 0.79 | 0.66 | 0.54 |
| S NFL, 4y mean | 0.76 | 0.63 | 0.52 |
| CSF NFL, BL | **0.81** | **0.85** | 0.73 |
| CSF NFL, 1y | **0.89** | **0.84** | 0.68 |
| CSF NFL, 2y | 0.79 | 0.73 | 0.67 |
| CSF NFL, 4y | 0.52 | 0.56 | 0.62 |
| CSF NFL, 2y mean | **0.87** | **0.88** | 0.75 |
| CSF NFL, 4y mean | **0.86** | **0.87** | 0.76 |
| CSF NFH, BL | 0.73 | 0.78 | 0.61 |
| CSF NFH, 1y | 0.75 | 0.72 | 0.54 |
| CSF NFH, 2y | 0.67 | 0.64 | 0.60 |
| CSF NFH, 2y mean | **0.80** | **0.82** | 0.61 |
| CSF GFAP, BL | 0.71 | 0.75 | 0.54 |
| CSF GFAP, 1y | 0.76 | 0.71 | 0.59 |
| CSF GFAP, 2y | 0.61 | 0.59 | 0.50 |
| CSF GFAP, 2y mean | 0.71 | 0.69 | 0.54 |
| CSF CHI3L1, BL | 0.75 | 0.69 | 0.65 |
| CSF CHI3L1, 1y | 0.69 | 0.62 | 0.66 |
| CSF CHI3L1, 2y | 0.57 | 0.51 | 0.51 |
| CSF CHI3L1, 2y mean | 0.70 | 0.62 | 0.60 |
| CSF MMP-9, BL | 0.72 | 0.68 | **0.83** |
| CSF MMP-9, 1y | 0.63 | 0.59 | 0.72 |
| CSF MMP-9, 2y | 0.53 | 0.52 | 0.67 |
| CSF MMP-9, 2y mean | 0.71 | 0.69 | **0.86** |
| CSF OPN, BL | 0.78 | 0.76 | 0.76 |
| CSF OPN, 1y | 0.57 | 0.59 | 0.74 |
| CSF OPN, 2y | 0.49 | 0.53 | 0.57 |
| CSF OPN, 2y mean | 0.65 | 0.66 | 0.72 |
| CSF CXCL1, BL | 0.73 | 0.74 | 0.69 |
| CSF CXCL1, 1y | 0.64 | 0.52 | 0.60 |
| CSF CXCL1, 2y | 0.47 | 0.42 | 0.46 |
| CSF CXCL1, 2y mean | 0.61 | 0.59 | 0.61 |
| CSF CXCL8, BL | 0.71 | 0.69 | 0.57 |
| CSF CXCL8, 1y | 0.53 | 0.50 | 0.35 |
| CSF CXCL8, 2y | 0.46 | 0.39 | 0.29 |
| CSF CXCL8, 2y mean | 0.56 | 0.50 | 0.35 |
| CSF CXCL10, BL | **0.84** | **0.82** | **0.82** |
| CSF CXCL10, 1y | 0.66 | 0.59 | 0.62 |
| CSF CXCL10, 2y | 0.60 | 0.61 | 0.58 |
| CSF CXCL10, 2y mean | 0.75 | 0.72 | 0.72 |
| CSF CXCL13, BL | 0.76 | 0.76 | **0.80** |
| CSF CXCL13, 1y | 0.63 | 0.61 | 0.71 |
| CSF CXCL13, 2y | 0.53 | 0.57 | 0.70 |
| CSF CXCL13, 2y mean | 0.73 | 0.74 | **0.85** |
| CSF CCL22, BL | 0.71 | 0.75 | **0.81** |
| CSF CCL22, 1y | 0.58 | 0.57 | 0.61 |
| CSF CCL22, 2y | 0.55 | 0.54 | 0.58 |
| CSF CCL22, 2y mean | 0.66 | 0.70 | 0.76 |
| AUC: area under curve (from receiver operating characteristic curve analysis); EDA-3/NEDA-3: evidence of disease activity-3/no evidence of disease activity-3; y: year; CSF: cerebrospinal fluid; S: serum; BL: baseline; 1y: one year; 2y: two years; 4y: four years; 2y mean: mean level over two years; 4y mean: mean level over four years. AUCs ≥0.80 in bold. | | | |
