# Supplementary material for: Neurofilament levels, disease activity and brain volume during follow-up in multiple sclerosis
Source: J Neuroinflammation. 2018 Jul 18;15:209. doi: 10.1186/s12974-018-1249-7 (PMC6052680; doi:10.1186/s12974-018-1249-7)
Supplement: Supplementary file 3 — Linear regression modeling details. (DOCX 12 kb) [file 12974_2018_1249_MOESM3_ESM.docx]

**Linear regression modelling indicates CHI3L1 as the strongest predictor of** **BPF decrease**

To identify the most important factors for prediction of BPF decrease, linear regression modelling of BPF decrease during four years of follow-up was performed with an initial model including baseline number of T2 lesions in brain MRI, baseline BPF, baseline age, baseline EDSS, baseline level of mononuclear cells in CSF and baseline CSF levels of CXCL1, CXCL8, CXCL10, CXCL13, CCL22, NFL, NFH, MMP-9, GFAP, CHI3L1 and OPN. This initial model was not significant (adjusted R^2^ 0.19, p=0.18). Successively removing the least significant variable until the model became significant (p≤0.01) yielded a model including age, EDSS, CSF-mononuclear cells, CXCL10, NFH, MMP-9, CHI3L1, BPF and T2 lesions at baseline (adjusted R^2^ 0.37, p=0.007). Successively removing the least significant variable yielded a peak adjusted R^2^ peak at 0.41 (p=0.001) for a model including age, CSF-mononuclear cells, NFH, MMP-9, CHI3L1 and T2 lesions at baseline. Then again successively removing the least significant variable aiming for a model in which all remaining variables were significant on their own left only baseline CHI3L1 in the model (R^2^ 0.26, p=0.001).

To identify the most important factors for prediction of new T2 lesions, linear regression modelling of number of new T2 lesions during four years of follow-up was performed with an initial model including baseline number of T2 lesions in brain MRI, baseline BPF, baseline age, baseline EDSS, baseline level of mononuclear cells in CSF and baseline CSF levels of CXCL1, CXCL8, CXCL10, CXCL13, CCL22, NFL, NFH, MMP-9, GFAP, CHI3L1 and OPN. This initial model was not significant (adjusted R^2^ -0.24, p=0.87). Successively removing the least significant variable aiming for a significant model failed. The significance level p≤0.01 could not be reached. When all variables except NFL, NFH and MMP-9 had been excluded from the model, adjusted R^2^ was 0.10 (p=0.09). Last remaining variable was MMP-9 (R^2^ 0.11, p=0.05).

To identify parameters associated with BPF decrease, linear regression modelling of BPF decrease during four years of follow-up was performed with an initial model including the variables that correlated significantly with BPF decrease in Table 3, *i.e.* baseline number of T2 lesions in brain MRI, one year S-NFL, one year CSF-NFL, baseline CHI3L1, one year CHI3L1, two year CHI3L1, mean CHI3L1 and baseline OPN. This initial model was not significant (adjusted R^2^ 0.25, p=0.02). Successively removing the least significant variable aiming for a significant model yielded a model including baseline T2 lesions, baseline OPN, CSF-NFL at one year and mean CHI3L1 (adjusted R^2^ 0.27, p=0.006). When again successively removing the least significant variable, adjusted R^2^ peaked with a model including baseline T2, baseline OPN and one year CHI3L1 (adjusted R^2^ 0.30, p=0.002) and then decreased (baseline T2 and one year CHI3L1 (adjusted R^2^ 0.29, p=0.001), only one year CHI3L1 (R^2^ 0.29, p=0.001)).

To identify parameters associated with new T2 lesions, linear regression modelling of number of new T2 lesions during four years of follow-up was performed with an initial model including the variables with significant correlations with new T2 lesions in Table 3, *i.e.* baseline CSF-NFL, one year CSF-NFL, mean CSF-NFL over two and four years, baseline CXCL13, mean CXCL13, mean CCL22 and mean MMP-9. This initial model was significant (adjusted R^2^ 0.34, p=0.008). Successively removing the least significant variable yielded peak adjusted R^2^ with a model including mean CCL22, one year CSF-NFL and baseline CXCL13 (adjusted R^2^ 0.40, p<0.001) and adjusted R^2^ then decreased (one year NFL and mean CCL22 (adjusted R^2^ 0.36, p<0.001), only one year CSF-NFL (R^2^ 0.32, p<0.001)).
